# To Crowdfund Research, Scientists Must Build an Audience for Their Work

**DOI:** 10.1371/journal.pone.0110329

**Published:** 2014-12-10

**Authors:** Jarrett E. K. Byrnes, Jai Ranganathan, Barbara L. E. Walker, Zen Faulkes

**Affiliations:** 1 Department of Biology, University of Massachusetts Boston, Boston, Massachusetts, 02125, United States of America; 2 National Center for Ecological Analysis and Synthesis, Santa Barbara, California, 93101, United States of America; 3 Institute for Social, Behavioral, and Economic Research, University of California Santa Barbara, Santa Barbara, California, 93106, United States of America; 4 Department of Biology, The University of Texas-Pan American, Edinburg, Texas, 78539, United States of America; Cinvestav-Merida, Mexico

## Abstract

As rates of traditional sources of scientific funding decline, scientists have become increasingly interested in crowdfunding as a means of bringing in new money for research. In fields where crowdfunding has become a major venue for fundraising such as the arts and technology, building an audience for one's work is key for successful crowdfunding. For science, to what extent does audience building, via engagement and outreach, increase a scientist's abilities to bring in money via crowdfunding? Here we report on an analysis of the #SciFund Challenge, a crowdfunding experiment in which 159 scientists attempted to crowdfund their research. Using data gathered from a survey of participants, internet metrics, and logs of project donations, we find that public engagement is the key to crowdfunding success. Building an audience or “fanbase” and actively engaging with that audience as well as seeking to broaden the reach of one's audience indirectly increases levels of funding. Audience size and effort interact to bring in more people to view a scientist's project proposal, leading to funding. We discuss how projects capable of raising levels of funds commensurate with traditional funding agencies will need to incorporate direct involvement of the public with science. We suggest that if scientists and research institutions wish to tap this new source of funds, they will need to encourage and reward activities that allow scientists to engage with the public.

## Introduction

### Rise of Science Crowdfunding and the Decline of Public Research Funding

Over the past five years, a new method of Internet-based fundraising known as crowdfunding has exploded in popularity [1]. In the first six months of 2013 alone, almost US$200 million was raised for technology and arts-related projects on just one leading crowdfunding website [2]. But what role can crowdfunding play in the sciences? How must science adapt to take advantage of this growing pool of available funding?

The rise of crowdfunding comes at a time when scientists are facing increasing competition for declining sources of public funding [3]. Between 1992 and 2012, state appropriations fell by 15% at the U.S. public research universities with the largest research and development funding inflows [4]. Further, U.S. federal funding for research in most physical sciences, mathematics, and engineering has declined or remained relatively flat in inflation-adjusted purchasing power for several decades [5]. A recent National Research Council report concluded that federal funding for university research has been unstable overall, and is declining in inflation adjusted dollars [6]. As one consequence, the average age of principal investigators receiving their first major research grant (R01) from the National Institutes of Health is 42 years old [7].

Interest in science crowdfunding is largely driven by this steady downturn in government funding for science, particularly in the United States. Indeed, well before crowdfunding began to catch on among scientists, Gaggioli and Riva [8] suggested “crowd-funding as a possible strategy to cope with the lack of investments in research, as well as to increase democratization in the sciences”. Crowdfunding democratizes science funding by using a model for supporting projects that charities have long used: aggregating small donations from many people to achieve a successfully fund a project. The arrival of dedicated Internet platforms truly democratized this fundraising model by removing the need for substantial infrastructure and manpower traditionally needed for charity fundraising. Crowdfunding now allows a wider range of potential users, including scientists, to ask for and receive small donations. These users then become involved in science by helping shape what projects get funded and by maintaining their personal investment in new fields of scientific inquiry.

Crowdfunding serves a further need beyond merely funding science. Crowdfunding provides a crucial conduit for communication between scientists and the public. To create a crowdfunding proposal, scientists must talk about their work in a way that appeals to people outside of the academy. They must be good science communicators, and then are rewarded for their efforts with money for their research.

### Theoretical Context: Crowdfunding and Science Communication

Little is understood about how crowdfunding works and whether the lessons of the science communication literature can provide a roadmap for successful efforts. The nascent literature on the entire field of crowdfunding is found primarily in popular journals and the blogosphere. Analysis of what drives successful campaigns are largely case studies of the most successful projects [9]. A small number of recent articles focus on crowdfunding within the context of new Securities and Exchange Commission regulations [10], [11], and opportunities for entrepreneurs and small businesses [1], [12], [13], [14].

The literature documents some best practices that have been gleaned through informal observations of crowdfunding websites. Hughes [15], for example, emphasizes the benefits of creating a fan base for your research through crowdfunding, which can lead to increased visibility and other opportunities down the line. Ordanini et al. [1] recognize the importance of family, friends, and extended social networks as the initial investor base for a successful crowdfunding campaign. Wheat et al. [16] focus on science crowdfunding and, in particular, discuss the process of how researchers run crowdfunding campaigns.

The advent of science crowdfunding also builds on recent trends in publicly engaged science communication. Drawing on [17], [18], we define science communication as activities that scientists engage in to communicate their research to various publics outside of the scientific community in order to build awareness, interest, and understanding. These activities increasingly include online and electronic public dissemination of science (e.g., [19], [20]). Across the disciplines in higher education there have been increasing calls for more publicly and socially engaged research agendas; scholarship that asks socially pertinent questions, science that incorporates the participation of the objects of science in experimental design (particularly in policy-relevant and health sciences); and science that is disseminated to and connects with the public in new ways [17], [21], [22], [23].

Yet, there are multiple obstacles to publicly engaged science communication. The values that underlie promotion and tenure in science do not often reward public engagement, and in fact public engagement can be costly to publication productivity and scientific reputation among peers [17], [24], [25], [26], [27], [28], [29], [30], [31], [32]. Similarly, traditional science review and funding mechanisms do not typically encourage (or pay for) public engagement, with the exception of the National Science Foundation Broader Impacts requirement [33], [34], [35], [36], [37]. Finally, there are great challenges for scientists to develop equitable languages and relationships with the public while balancing scientific and community objectives [38], [39], [40], [41], [42], [43].

This study contributes to these literatures by systematically illustrating the important links between science communication, public engagement, and the burgeoning crowdfunding phenomenon. Given recent stagnation in the availability of science research funding, publicly engaged science communication may become a more attractive option if it results in funding resources.

### Successful Science Crowdfunding: What Does It Take?

In fields where crowdfunding is now a significant source of funds, such as in the arts and technology, it took 3–5 years before participants were able to successfully fund projects in range of hundreds of thousands to millions of dollars [44]. This raises the question: what steps must individual researchers and research institutions take to develop the ability to leverage these large amounts of funds for science?

Successful crowdfunding relies on broad appeal and engagement with a large audience. Examples of this dependence can be seen from a leading crowdfunding site where many projects in 2012 raised over a million dollars [44]. Many of the most successful projects come from artists with huge fanbases (e.g., musician Amanda Palmer, who set a crowdfunding record for music [9], has over a million followers on Twitter; https://twitter.com/amandapalmer) or for extensions of extremely popular products with a built-in audience (e.g., a watch for smartphones [45] or sequels to the Ultima video games [46]). The same dynamic between audience size and crowdfunding success appears to hold for science. For example, the British charity Cancer Research UK routinely raises over £50,000 for individual research projects via crowdfunding (Table S1). Cancer Research UK and its predecessor organizations have spent decades building an audience for their work. It follows that their success in research crowdfunding stems from leveraging an extensive existing donor base. As with Cancer Research UK, the individuals behind these projects have built large audiences for their work over many years [9]. These examples suggest that building an engaged online audience through outreach by scientists is key to successful crowdfunding for research.

While attitudes among most scientists towards outreach and engagement are unenthusiastic [47], the last decade has witnessed dramatic growth in the visibility of scientists online [48]. Scientists are increasingly communicating their work to a public audience via online means like blogs and Twitter [49], [50], [51]. We therefore set out to ask how the amount of money one could raise via crowdfunding is influenced by: 1) building an audience for one's work via science communication, 2) the amount of effort put into communicating one's science, and 3) the different avenues one used to communicate their work.

To explore the potential link between online science engagement and successful crowdfunding, we organized a crowdfunding for science initiative, the #SciFund Challenge (hereafter #SciFund). We set up #SciFund with standardized conditions for participants, such as project duration, so that we could use the data to investigate the factors influencing proposal success. We collected data from patterns of web traffic, metrics from social media websites (e.g., Facebook and Twitter), donations, and from a survey of participating scientists. We used these data for an analysis of the principles of crowdfunding success using a series of statistical models. With well over a hundred crowdfunding projects taking place under the auspices of #SciFund, this study is the most comprehensive analysis of science crowdfunding to date. Here we provide results from #SciFund to demonstrate the link between online outreach and success in crowdfunding for research dollars.

## Methods

### Structure of the #SciFund Challenge

#SciFund is a crowdfunding experiment for science. As part of #SciFund, we organized scientists to run their own crowdfunding projects simultaneously for their research under the #SciFund banner. #SciFund ran in a round-based format, with three rounds occurring between July 2011 and December 2012. Each round lasted several months and was divided into three phases: (1) soliciting proposals, (2) training participants, and (3) executing proposal “campaigns”. In the soliciting phase of each round, #SciFund organizers encouraged scientists (across disciplines and countries) to participate in this crowdfunding exercise, via e-mail lists, blog posts, and social media (e.g., Twitter and Facebook). This soliciting phase lasted three months in the first round and one month each for the next two rounds. To ensure scientific credibility, each scientist who signed up to participate was vetted, via an application form that was evaluated by a science advisory board consisting of experts in biology, physics, chemistry, and sociology (at least two scientists who deemed their experience relevant to the project evaluated every application). In the training phase of each round, organizers trained the scientists to run a crowdfunding campaign via instructional blog posts on our website (round 1: http://scifund.wordpress.com; afterwards: http://scifundchallenge.org, all posts are still present and used regularly for new rounds), an online discussion group, and by encouraging discussion and feedback on draft projects and project videos within a private online space. This training phase lasted one month in each round. By the end of the training phase, participants had a fully formed crowdfunding proposal ready to be deployed.

In the executing phase of each round, the #SciFund crowdfunding projects and any accompanying videos went “live” on the Internet. All projects within a round launched simultaneously and ran for the same length of time. Although all #SciFund projects were running under the same banner, each participating scientist fundraised primarily for his or her own project (that is, there was no collective fundraising, although during the campaign periods, the project organizers advertised and promoted the #SciFund Challenge more broadly). Most projects each had a single scientist behind them, but there were several multi-researcher projects in each round. A wide range of scientific disciplines were represented (Table 1), although most projects focused on ecology or conservation biology, reflecting the professional networks of the #SciFund organizers. The total number of projects and the number of days of fundraising varied with each round (33–45 days, see Table 2).

**Table 1:** Distribution of #SciFund crowdfunding projects (across rounds) by academic discipline.

Academic discipline	Number of #SciFund projects across rounds
Conservation biology and ecology	100
Psychology	8
Biomedical research	6
Organic chemistry	6
Human development	5
Evolution	4
STEM education	4
Climate science	3
Computer science	3
Genetics	3
Anthropology	2
Applied math	2
Open science	2
Astronomy	1
Business research	1
Cancer biology	1
Engineering	1
Neuroscience	1
Paleontology	1
Political science	1
Seismology	1
Toxicology	1

**Table 2:** Descriptive summary statistics about duration and project performance from all three rounds of the #SciFund Challenge.

Round	Dates	Days	Projects	Projects funded at 100%	Percent funded at 100%	Total raised	Project Average	Project Median
1	Nov. 1–Dec. 15, 2011	45	49	10	20.40%	$76,230	$1,555.71	$1,104.00
2	May 1–May 31, 2012	31	75	33	44.00%	$100,345	$1,341.37	$1,046.00
3	Nov 11–Dec. 15, 2012	33	35	16	45.70%	$75,978	$2,170.80	$1,440.00
Overall			159	59	37.10%	$252,811		

These projects were hosted on a special section of the crowdfunding platform RocketHub (http://scifund.rockethub.com). Resulting funds were directly disbursed by RocketHub to the recipients designated by the participants (generally the participant's home institution or affiliated nonprofits). The only charges that #SciFund participants incurred were RocketHub's customary fees for crowdfunding projects running on their site (8–12% of the total raised, depending on whether they achieved their funding goal). #SciFund participants received funds even if they did not reach their financial targets, unlike the funding model for some crowdfunding platforms, where funds are disbursed only if the project is fully funded. It should be noted that several of this paper's authors (Walker, Byrnes, and Faulkes) ran individual crowdfunding projects under the #SciFund banner in round one. The organizers of #SciFund were not paid by RocketHub nor did they receive any funds, either directly or indirectly, from #SciFund participants or donors (other than the donor funds Walker, Byrnes, and Faulkes received from their individual projects).

### Data Sources

After each of the three #SciFund rounds, we compiled data from three sources to analyze the factors that led to successful crowdfunded projects. First, we acquired the web visit and donation logs of each project from RocketHub. Second, we collected publicly available information from the Internet. Each RocketHub project page included buttons allowing visitors to tweet about the project on Twitter (http://twitter.com), or “Like” the project on Facebook (http://facebook.com). The number of tweets and “Likes” were publically displayed on the project page, were updated dynamically, in real time. We recorded the number of tweets and “Likes” from each #SciFund project page within hours of the campaign ending. Thus, these are conservative measures of project promotion for these two social media sites, because they only include button clicks on the RocketHub page, and not tweets or “Likes” created by other means (e.g., copying the project URL directly). Similarly, project videos were embedded on the RocketHub project pages, but hosted by other websites (e.g., http://youtube.com, http://vimeo.com), which also displayed the number of video views publically, and updated the numbers in real time. The number of times project videos were viewed was also collected within hours of the campaigns ending [52], [53], [54].

Last, we designed a survey for all #SciFund participants to measure: (1) strategies used to create crowdfunding materials, (2) strategies used to promote crowdfunding campaigns, (3) social network size (i.e., number of Facebook friends and Twitter followers), and (4) various aspects of ongoing online outreach activities (e.g., Do they have a blog?); see Table S2 for a complete list of questions. This survey was completed by #SciFund participants in the first few weeks after their crowdfunding project finished. The survey was answered by 47 of the 49 #SciFund round one participants, 48 of 75 round two participants, and 22 of 35 round three participants. The survey instrument for rounds two and three differed in some ways from the instrument we used for round one. Specifically, we changed the requested response for several questions from a Likert scale selection to a specific quantitative answer (see Table S2 for complete list of changes). For example, questions regarding the number of tweets, Facebook posts, Google+ posts, and e-mails made by participants required a numerical response in the survey instruments for rounds two and three (where they had required a Likert scale selection in the round one survey). We asked about number of hours spent promoting a project, but found that these self-reported numbers proved unreliable and were often answered qualitatively rather than quantitatively in the survey.

In addition to quantitative data, the surveys asked opened-ended questions that collected qualitative data about participants' experiences during the #SciFund Challenge, such as what types of outreach and engagement they thought were most and least effective in their campaigns, and overall satisfaction with the experience. These data were compared to the statistical models to determine if participant perceptions about crowdfunding success and failure matched the results of the statistical models.

### Factors Influencing Success of #SciFund Projects

To determine the chain of events that attracted donations for the #SciFund projects, we explored four questions using statistical modeling with the data from round one. We then took the fit models, and challenged them with the data from rounds two and three to verify their conclusions. The questions were: First, what effect did the number of donors have on crowdfunding success? Second, where were donations coming from? That is, were donations merely due to scientists somehow drawing attention to their projects, or did personal connections generated through online social networks play a role? Third, was the attention a project received generated from existing social networks or other forms of “buzz” generated by the #SciFund campaign itself? Fourth, did long-term scientific outreach via blogging increase scientists' outreach-generated social networks? Thus, we hoped to examine the influence of a scientist's public presence on crowdfunding success.

As we were dealing with count data in many of the analyses, most data were modeled using generalized linear models with linear or log links [55] and a quasi-Poisson error distribution to account for over dispersion [56]. All models were fit using the base package in R Version 2.14.2 [57]. To examine the amount of variance in the response variables retained by our statistical models, we calculated the R^2^ of the relationship between predicted and observed values of response variables [58]. Note that different pieces of the analysis had different sample sizes depending on whether survey respondents included answers or not. Sample sizes are reported with each analysis.

To examine the relationship between number of donors and total amount raised, we fit a linear relationship as described, but set the intercept at zero, as zero contributions meant zero dollars were raised by definition. We hypothesized that several factors could influence the total number of contributors and fit a model accordingly. First, the number of times a project was viewed should directly influence the number of contributors. Because projects had clear financial goals, and because the probability of someone viewing a project after it hit its funding goal may change, we separated pre- and post-goal page views. Second, the size of someone's personal social network may influence the number of contributors, as friends and family may be more likely to donate to a project. Last, the size of a scientist's online social network generated by previous online outreach activities may also influence the total number of contributors; this was measured by number of Twitter followers.

For this and other analyses incorporating project page views, we excluded a single outlier. One project had an enormous number of project page views: 38,131, compared to the mean of 2,217.75 and median of 1,070. The next highest number of page views was 6,702. The number of page views in the most viewed project was due to promotion on two highly popular web sites that other projects did not have. This outlier exerted an enormous leverage on the analysis and was therefore excluded. Analyses with this outlier project were qualitatively the same, but quantitative results and amount of variance retained were quite different. In analyses of future rounds, should there be a larger sample size in the 7,000–30,000-page-view range, we would be better able to detect linear or nonlinear relationships involving this data point. For this round, the 38,131 data point was excluded for analyses involving page views.

We next evaluated the relationship between page views and three predictors of project popularity: the size of one's social network (Facebook friends), the size of their outreach generated social network (Twitter followers), and the ability of a scientist to cultivate interest in a project as measured by the number of people who had clicked the “Like on Facebook” button on a project's web page. Again, we split pre- and post-goal views. For pre-goal project page views, we fit a model as above. For post-goal project page views, we only analyzed the subset of projects that met their goal. Additionally, a number of projects met their goal during the final days of #SciFund. Most of these projects had no post-goal project page views. We therefore fit a model with a log rather than linear link function.

Last, to explore whether ongoing online outreach efforts by scientists increased their Twitter followers, we looked at the relationship between Twitter followers and the average number of monthly blog posts by #SciFund scientists who had established blogs. We assumed the direction of causality went from monthly blog posts to number of Twitter followers, because it seemed unlikely that researchers would blog more often because they had more Twitter followers. Rather, we hypothesized that the more frequently a researcher posted to their blog, the more likely they would be to attract a larger following on Twitter. For participants who did not have a blog, we set their number of monthly posts to 0. The age of these blogs ranged from a few months to nearly ten years. As blog age and posting frequency were highly correlated (r = 0.68), we did not include them as independent measures of online outreach.

### The Role of Effort

After re-evaluating the models fit during round one with round two and three data, we noted a discrepancy in the link between audience size and number of page views (see Results). We also noted that the difference in effectiveness of pre- versus post-goal page views was much weaker. We therefore revised several questions in our survey in order to better assess participant effort for rounds two and three. We were thus able to ask, how does effort modify the effect of audience size on the ability of a researcher to bring people to view their project? For this model, we looked at audience size and number of posts on Twitter and Google+ as well as how the two interacted. We also estimated parameters for the effect of number of people contacted via email and the number of people contacted by project scientists in the press. We fit models with a Gaussian error term, as the data did not meet the assumption of a mean-variance scaling relationship from a Poisson or quasi-Poisson error distribution. We removed one outlier data point, as its number of press contacted was two orders of magnitude larger than any other data point, and was likely a typo on the form or a misunderstanding of the question (post-hoc requests for verification from the participant yielded no response). We fit this model both for total page views and pre- and post- goal page views. However, due to the smaller sample size for post-goal page views (27) and the high number of parameters for the model (k = 10), we elected to drop the parameters assessing the impact of Google+, as they were not different from 0 in the initial model and contributed to an exceedingly high variance inflation factor in the post-goal page views model. Last, we fit a simple model examining to what extent post-goal page views were merely explained by pre-goal page views, as none of our predictors appeared to explain variability adequately. After analysis of our increased sample size, we also recognized that Facebook “Likes” are often an epiphenomenon of people visiting projects, not a causal driver. Indeed, they were highly correlated with variables that were more causally related to effort, such as number of press contacted (r = 0.76), number of Tweets (r = 0.61) or number of Facebook posts (r = 0.81).

## Results

### Money Raised through the #SciFund Challenge

**Figure 1:**
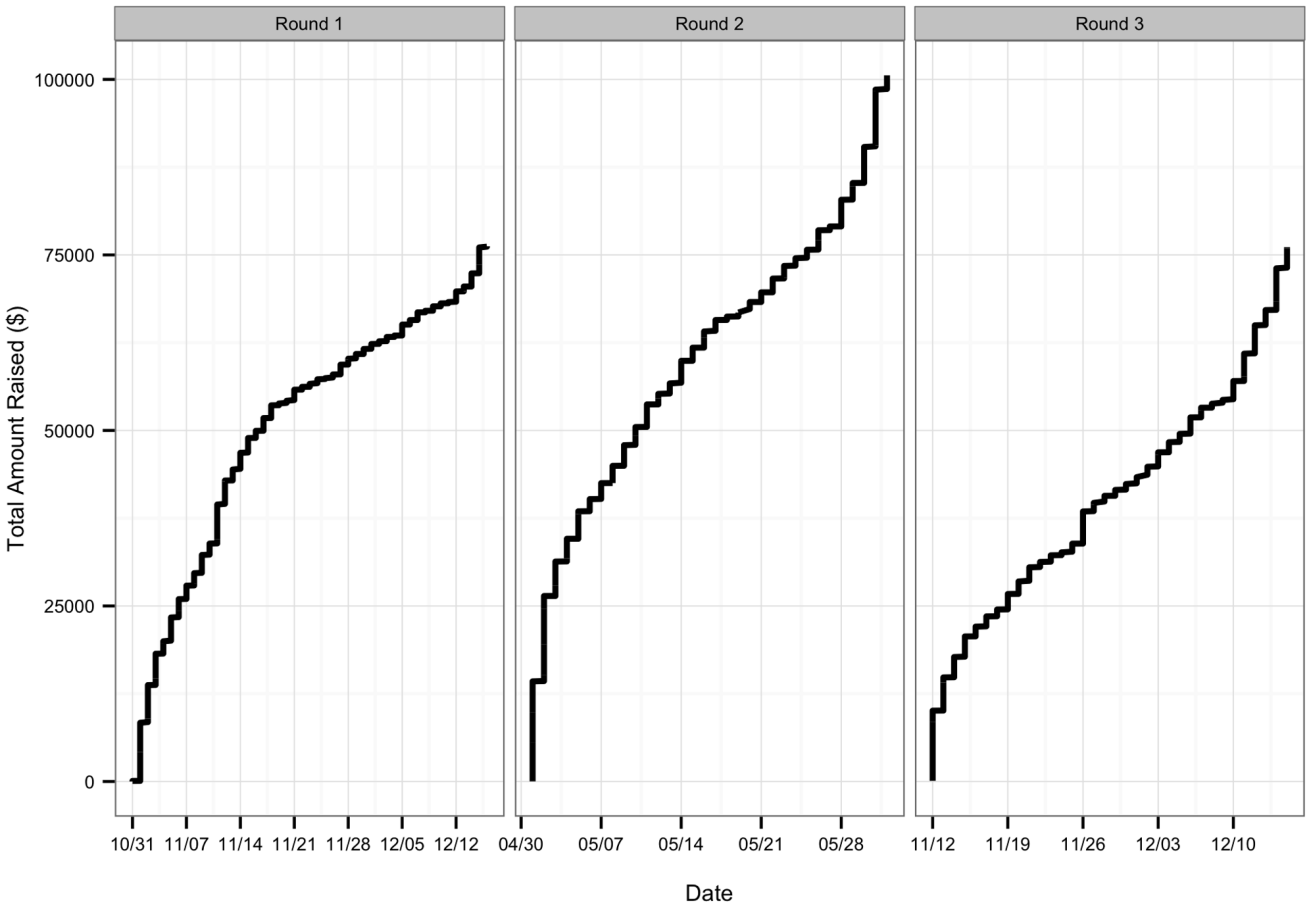
Crowdfunding donation patterns. The daily time series of donations during the firth three rounds of #SciFund.

Over three rounds, #SciFund raised US$252,811 from 3,904 donors funding 159 projects (see Table 2 for summary statistics). The timing of donations was relatively similar for all three rounds and conformed to what has been observed in other crowdfunding campaigns [59]: a large amount of funds raised early in the campaign, a gradual leveling out, and then a sudden burst of funding activity at the end (Fig. 1).

The first round of #SciFund raised US$76,230 over 45 days from at least 1,195 donors (donor counts for rounds one and two are likely to be underestimates, as donor names in those rounds were used to identify unique donors and multiple donors may have had the same name). There was a large range in the financial targets of the 49 #SciFund projects (range: US$500–20,000; median: US$3,500; average: US$4,601). Similarly, there was a large range in the amount received by the projects, as measured by total dollars (range: US$122–10,171; median: US$1,104; average: US$1,556). The project that raised the most, both in terms of dollars raised and percentage of goal (US$10,171 raised on a US$6,000 goal, 170% of target fundraised), was an outlier, as the second-highest amount fundraised was less than half of the first-place take (US$5,085). Ten projects matched or exceeded their targets (20% of projects); all six projects that asked for US$1,200 dollars or less met or exceeded their target.

Round two's 75 projects raised US$100,345 over the course of 31 days with 44% of participants achieving or exceeding their funding goal. At least 1,579 donors contributed to round two (likely an underestimate, as with round one, due to shared donor names). The financial targets of round two projects tended to be much lower than for round one and the range of dollar targets was also narrowed (range: US$333–12,000; median: US$2,000; average: US$2,215). A major reason for these lower funding goals was that #SciFund organizers, based on round one experience, strongly recommended that round two participants lower their financial targets. The amounts raised in round two were within a tighter band than in round one, but the median amount raised remained relatively steady (range: US$30–5,688; median: US$1,046; average: US$1,341).

Round three's 35 projects raised US$75,978 over 33 days with 46% of projects achieving or exceeding their goal. Round three had contributions from 1,130 donors (an exact count, unlike with rounds one and two). The financial targets of round three projects generally rose from the levels found for round two, though they were still lower than the targets for round one (range: US$380–10,000; median: US$2,500; average: US$3,083). In terms of the amounts actually raised, round three projects were on average the most successful of the three rounds (range: US$0–8,645; median: US$1,476; average: US$2,177). This is likely because the training that the Round 3 participants received was refined based on Rounds 1 and 2, and thus more accurate and effective.

### Exploratory Modeling of Factors Influencing Success of Round One #SciFund Projects

**Figure 2:**
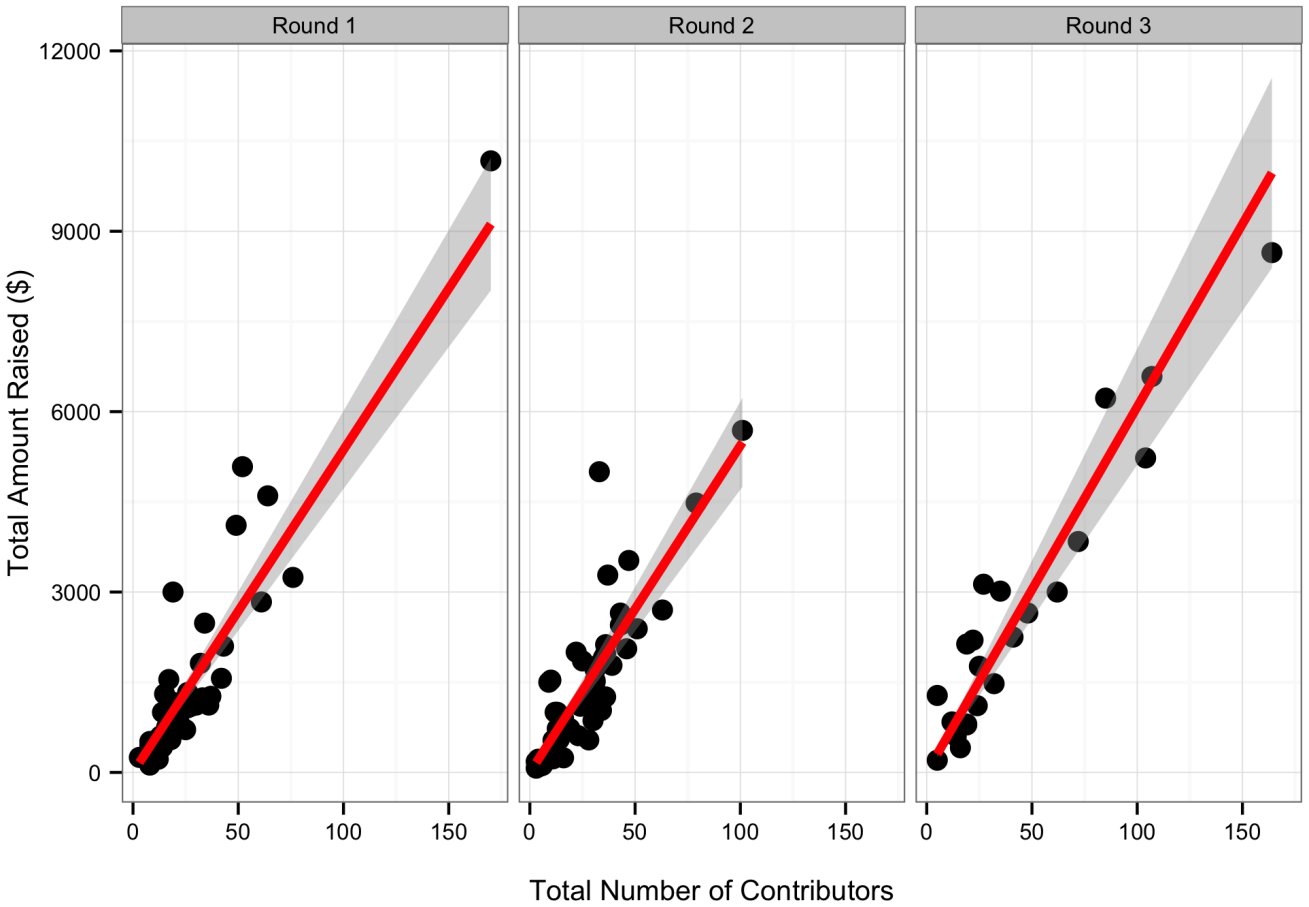
Total dollars raised plotted against the number of contributors. Line represents best fit from model described in the text. Shaded grey area represents the 95% confidence interval around the fit relationship.

**Figure 3:**
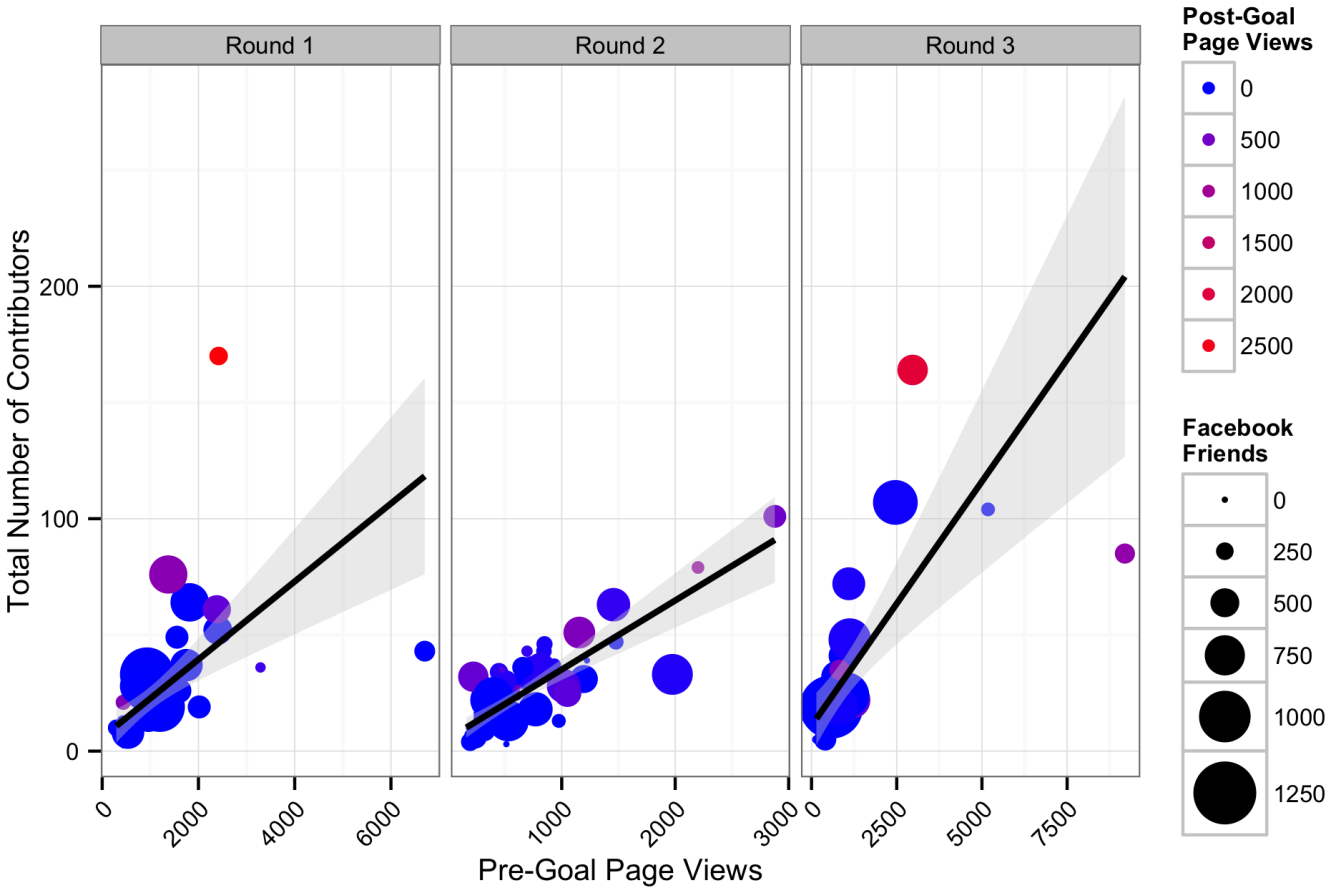
Factors affecting number of contributors to a project. Plot shows the number of contributors plotted against the number of Facebook friends. Size of points shows the number of page views before achieving success. Color shows the number of project page views after goals were reached with blue representing no views to red representing many views. Line represents best fit from generalized linear model between x and y. Shaded grey area represents the 95% confidence interval around the fit relationship.

Overall, in our exploratory analysis for round one, we found a relationship between online outreach efforts and funding. The number of contributors influenced total amount raised (Fig. 2, Likelihood Ratio χ^2^ = 567.95, DF = 1, p<0.001, n = 47): for every contributor, projects raised a mean of US$54.19 (S.E. = 3.19). 86.9% of the variance in money raised was retained by the model. The number of Facebook friends and page views, both before and after a project goal was reached, influenced total number of contributors (Table 3 and 4, n = 30, Fig. 3). The number of Twitter followers, however, did not. 85.3% of the variation in number of contributors was retained by the model. Before a project hit its initial goal, an average of 108 views was needed to generate one contribution. After a project hit its goal, only 21 page views were necessary to generate an additional contributor. Projects had one contributor for every 53 Facebook friends the research had.

**Table 3:** Likelihood ratio tests evaluating predictors of number of contributors in round 1.

	LR χ^2^	Df	Pr(>χ^2^)
Twitter Followers	0.041	1	0.84
Facebook Friends	5.397	1	0.02
Pre-Goal Page Views	12.849	1	>0.001
Post-Goal Page Views	44.601	1	>0.001

**Table 4:** Coefficient estimates, standard errors, and t-tests of predictors in analyses of number of contributors in round 1.

	Estimate	Std. Error	t value	Pr(>|t|)
(Intercept)	4.497	3.925	1.146	0.263
Twitter Followers	−0.001	0.006	−0.224	0.825
Facebook Friends	0.019	0.008	2.301	0.03
Pre-Goal Page Views	0.009	0.003	3.544	0.002
Post-Goal Page Views	0.048	0.009	5.139	>0.001

**Figure 4:**
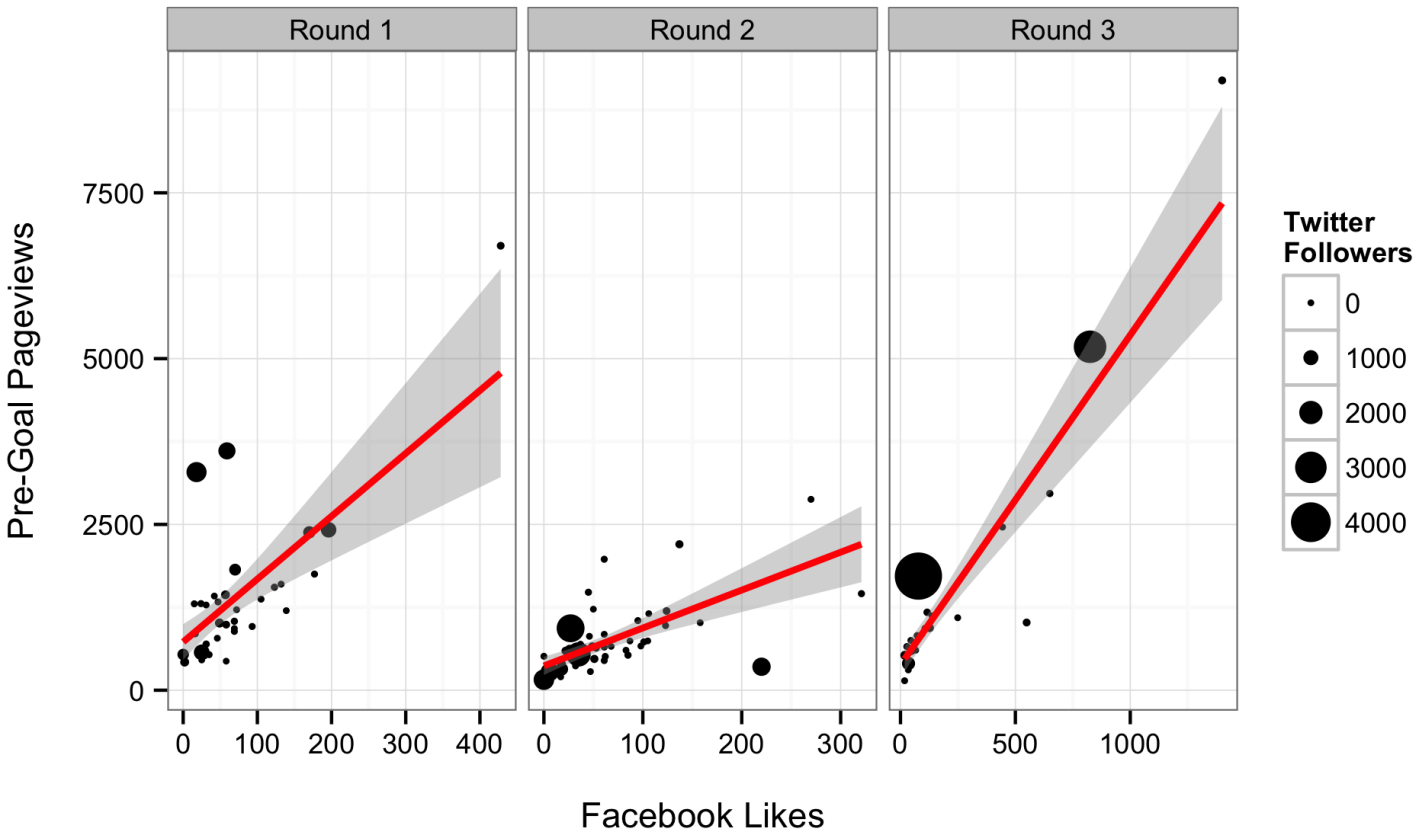
Relationship between Facebook “Likes”, number of Twitter followers, and project page views before a project hit its goal. Line represents best fit from model described in the text. Shaded grey area represents the 95% confidence interval around the fit relationship. Point size is proportional to the number of Twitter followers.

**Figure 5:**
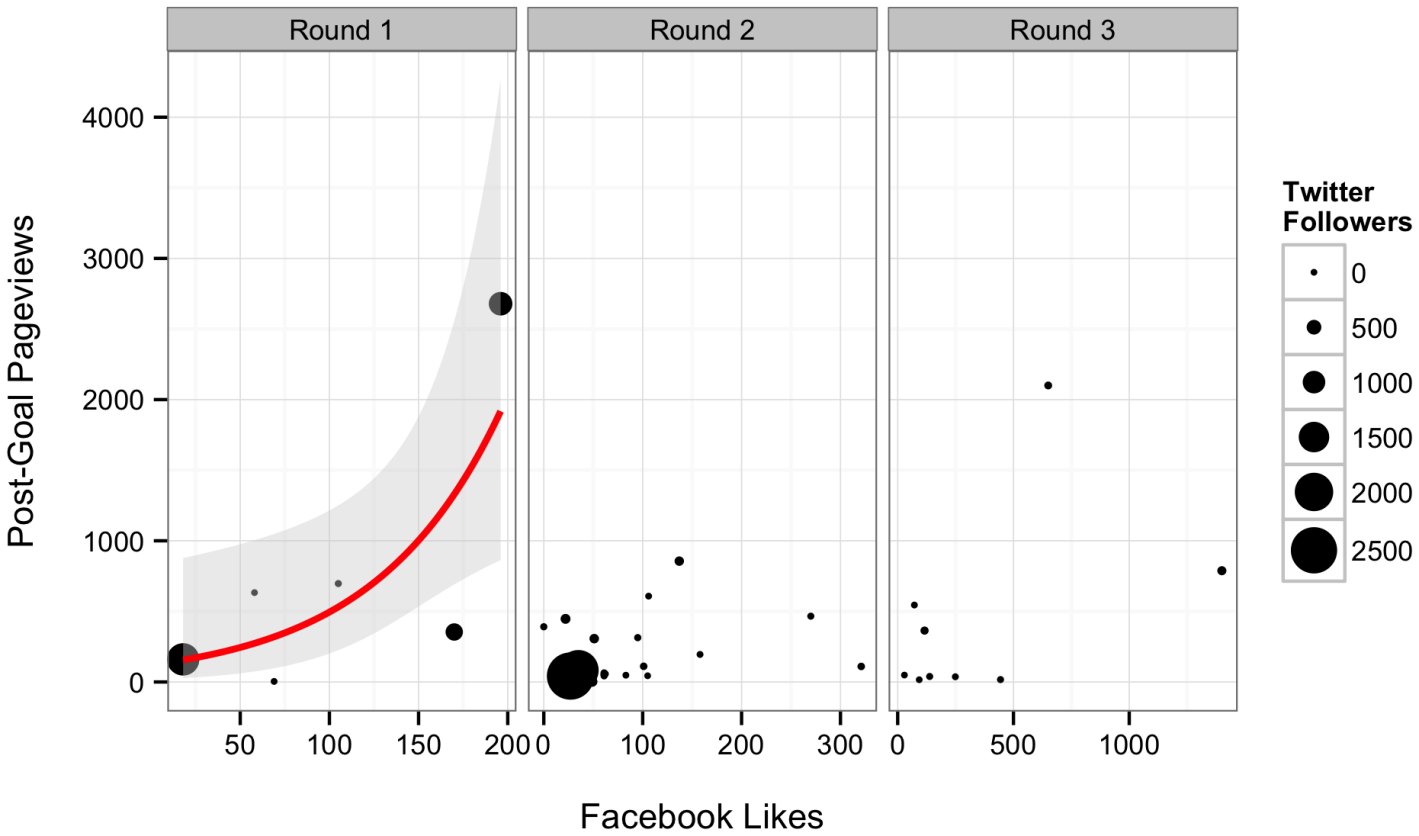
Relationship between Facebook “Likes” and the number of page views after a project has achieved its funding goal. Line represents best fit from model described in the text. Shaded grey area represents one standard error around the fit relationship.

Both Twitter followers and Facebook “Likes” influenced the number of project page views before reaching a goal (Table 5 and 6, n = 30, Fig. 4). Projects received a mean of 0.78 (S.E. = 0.28) page views per follower. They also received roughly 10 additional page views per Facebook “Like.” 78.3% of the variation in post-goal page views was retained in this model. For projects that met their goal, only Facebook “Likes” appeared to influence the number of page views (Table 5 and 6, n = 7, Fig. 5). This model retained 83.7% of the variation in post-goal page views.

**Table 5:** Likelihood ratio tests evaluating predictors of pre- (a) and post-goal page views (b) in round 1.

		LR χ^2^	Df	Pr(>χ^2^)
(a)	Twitter Followers	11.621	1	0.001
	Facebook Friends	0.97	1	0.325
	Facebook Likes	58.85	1	>0.001
(b)	Twitter Followers	0.307	1	0.579
	Facebook Friends	1.463	1	0.226
	Facebook Likes	8.466	1	0.004

**Table 6:** Coefficient estimates, standard errors, and t-tests of predictors in analyses of pre- (a) and post-goal page views (b) in round 1.

		Estimate	Std. Error	t value	Pr(>|t|)
(a)	(Intercept)	528.414	165.058	3.201	0.004
	Twitter Followers	0.782	0.284	2.752	0.011
	Facebook Friends	−0.345	0.355	−0.971	0.34
	Facebook Likes	10.04	1.769	5.675	>0.001
(b)	(Intercept)	5.674	1.147	4.949	0.016
	Twitter Followers	−0.001	0.001	−0.503	0.649
	Facebook Friends	−0.003	0.002	−1.114	0.346
	Facebook Likes	0.018	0.009	1.925	0.15

**Figure 6:**
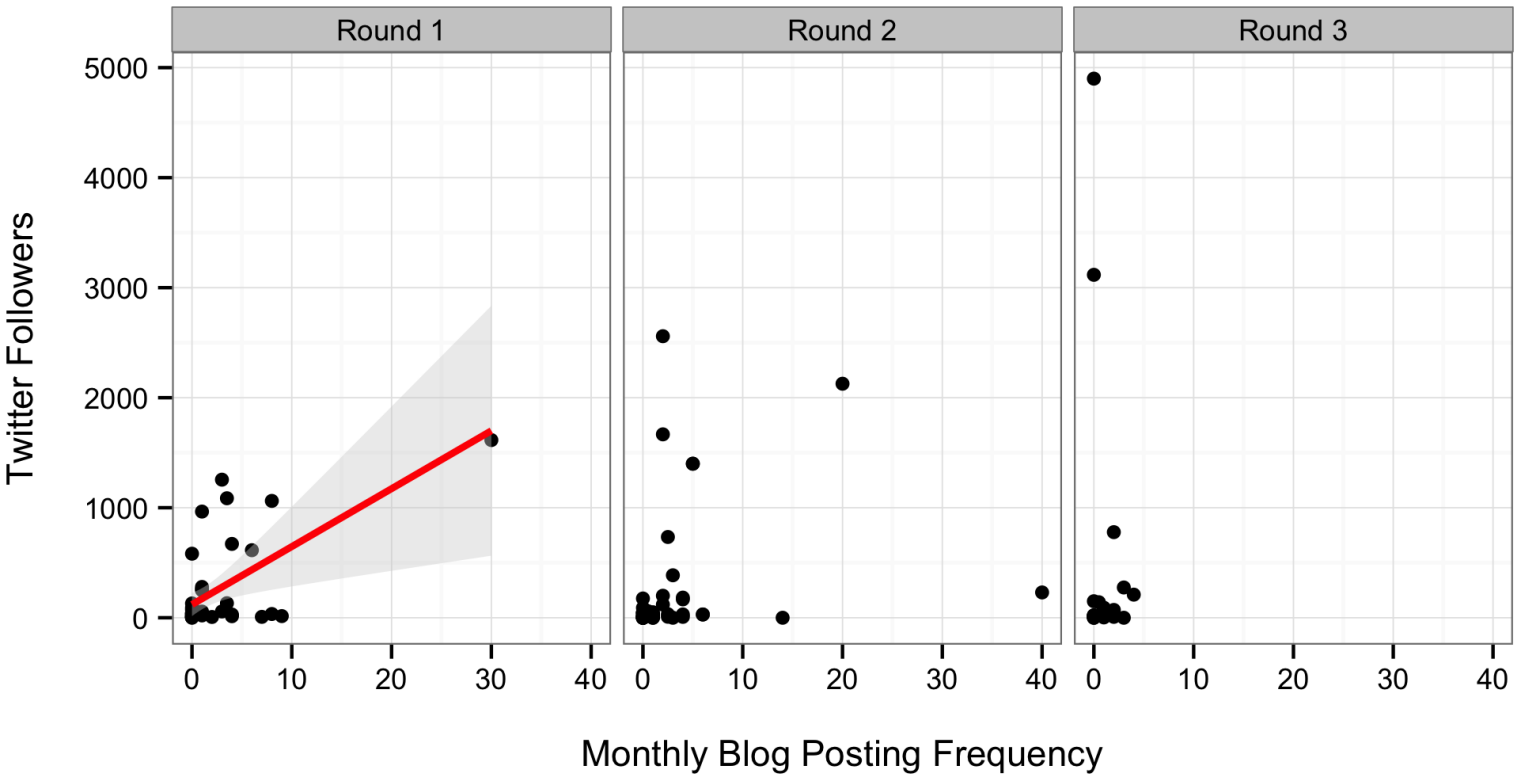
Relationship between monthly blog posts and number of Twitter followers. Line represents best fit from model described in the text. Shaded grey area represents on standard error around the fit relationship.

Posting frequency predicted Twitter followers (Fig. 6, Likelihood Ratio χ^2^ = 10.944, DF = 1, p<0.001, n = 35). For every monthly post, participants picked up a mean of 52.66 (S.E. = 19.96) additional followers. Only 34.4% of the variation in number of Twitter followers was retained by the model. Thus, we suggest that there are additional factors not quantified by our survey instrument that led to scientists aggregating an online following.

### Confirmatory Model of Factors Influencing Success of Rounds Two and Three #SciFund Projects

The broad message of the model from round one—that engaging audiences aided in funding—was retained in our analysis of further rounds. However, we found several discrepancies that were not supported in our confirmatory model analysis. Furthermore, our analysis of rounds two and three revealed a substantial role for effort. Overall, we find that effort on multiple fronts to engage a large audience was important for crowdfunding success. We found that the model suggested by the round one analysis held only insofar as dollars were linked to contributors (Slope = 57.04±2.96 SE, t = 19.29, p<0.001, R^2^ = 0.83) which in turn was determined by page views and weak support for Facebook network size (Table 7 and 8). The slope of the pre- and post-goal page view relationship with number of contributors had weak support for being different from one another (pre slope = 0.018±0.003, post slope = 0.037±0.010, t-test for difference t = 1.82, DF = 66, p = 0.07). However, both pre- and post- goal page views had no relationship with Twitter network size when using models developed from round 1 (p>0.50 for both). Clearly, the models we developed for project page views in round one did not hold for round two or three.

**Table 7:** Likelihood ratio tests evaluating predictors of number of contributors in rounds 2 and 3.

	LR χ^2^	Df	Pr(>χ^2^)
Facebook Friends	2.981	1	0.084
Pre-Goal Page Views	58.206	1	>0.001
Post-Goal Page Views	17.797	1	>0.001

**Table 8:** Coefficient estimates, standard errors, and t-tests of predictors in analyses of number of contributors in rounds 2 and 3.

	Estimate	Std. Error	t value	Pr(>|t|)
(Intercept)	6.523	2.504	2.605	0.011
Facebook Friends	0.011	0.006	1.816	0.074
Pre-Goal Page Views	0.018	0.003	6.524	>0.001
Post-Goal Page Views	0.036	0.01	3.64	0.001

### The Role of Effort

**Figure 7:**
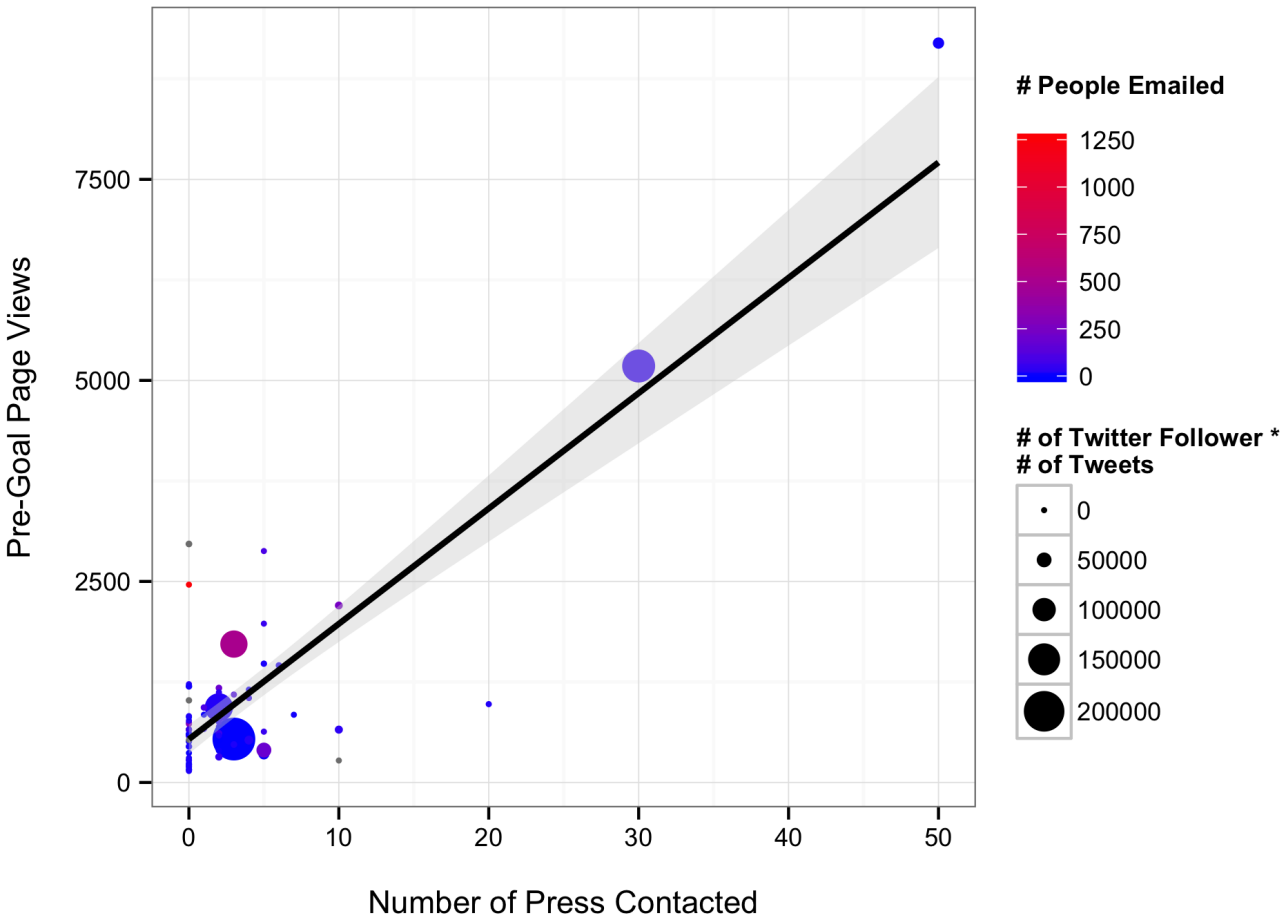
Relationship between pre-goal page views, press contacts, number of people emailed, and effort times engagement on Twitter. Line represents best fit from model between press and pre-goal page views. Shaded grey area represents the 95% confidence interval around the fit relationship.

Our initial hypotheses had anticipated that both effort on the part of a researcher and their network size should contribute to the success of their project. Our models incorporating effort (Table 9 and 10, Figs. 7, S4) demonstrated that contacting people via email is extremely effective with 1.72 visits per person emailed pre-goal. Pre-goal page views were also enhanced by number of press contact (∼93 page views per press contacted). Intriguingly, there was an interaction between Twitter network size and number of tweets, such that for every ∼75 followers, 1 tweet would bring in 1 page view. Assuming each click is an independent person, thus two tweets a day would ensure that roughly 80% of a scientist's Twitter network has viewed their project. Overall, our effort model provided modest explanatory power for pre-goal page views (R^2^ = 0.67). Post-goal page views seemed to be relatively uninfluenced by all factors (Table 9b). Instead, a simple model where post-goal page views was explained by pre-goal page views (i.e., a popular project continues to be popular) appears to provide some explanation for post-goal page views (LR χ^2^ = 7.09, DF = 1, p = 0.008, slope = 0.113±0.047 SE, intercept = 118.283±88.942 SE, R^2^ = 0.20).

**Table 9:** Likelihood ratio tests evaluating predictors of pre- (a,) and post-goal page views (b, c) in rounds 2 and 3.

		LR χ^2^	Df	Pr(>χ^2^)
(a)	Google+ Followers	0.118	1	0.731
	# of Google+ Posts	3.198	1	0.074
	# of Twitter Followers	2.432	1	0.119
	# of Tweets	0.189	1	0.663
	# of People Contacted by Email	21.47	1	>0.001
	# of Press Contacted	33.88	1	>0.001
	Google+ Followers * Posts	0.12	1	0.729
	# of Twitter Followers * Tweets	5.394	1	0.02
(b)	# of Twitter Followers	0.839	1	0.36
	# of Tweets	0.348	1	0.555
	# of People Contacted by Email	0.072	1	0.788
	# of Press Contacted	0.342	1	0.558
	# of Twitter Followers * Tweets	0.249	1	0.618
(c)	Pre-Goal Page Views	7.096	1	0.008

**Table 10:** Coefficient estimates, standard errors, and t-tests of predictors in analyses of pre- (a, d) and post-goal page views (b, c) in rounds 2 and 3.

		Estimate	Std. Error	t value	Pr(>|t|)
(a)	(Intercept)	572.711	93.726	6.11	>0.001
	Google+ Followers	0.003	0.097	0.028	0.978
	# of Google+ Posts	−14.324	11.371	−1.26	0.214
	# of Twitter Followers	−0.269	0.199	−1.354	0.182
	# of Tweets	−5.025	4.06	−1.238	0.221
	# of People Contacted by Email	1.72	0.371	4.634	>0.001
	# of Press Contacted	92.645	15.917	5.821	>0.001
	Google+ Followers * Posts	−0.001	0.002	−0.347	0.73
	# of Twitter Followers * Tweets	0.014	0.006	2.323	0.024
(b)	(Intercept)	156.213	57.159	2.733	0.012
	# of Twitter Followers	0.005	0.21	0.023	0.982
	# of Tweets	2.04	2.732	0.747	0.463
	# of People Contacted by Email	−0.05	0.188	−0.268	0.791
	# of Press Contacted	6.263	10.703	0.585	0.564
	# of Twitter Followers * Tweets	−0.002	0.003	−0.499	0.623
(c)	(Intercept)	118.283	88.943	1.33	0.194
	Pre-Goal Page Views	0.114	0.043	2.664	0.012

### Researcher Impressions of what Contributed to Success and Failure

In the survey, participants were asked about their impressions of “what worked” and “what did not work” to make their crowdfunding campaigns successful (see Table S2 for question list). Answers were open-ended, and several participants identified multiple factors in their answers. Overall, 14 reasons were identified for what worked (Table 11), and 15 for what did not work (Table 12). For the most part, participants' opinions about the sources of their crowdfunding success matched the outcomes of the statistical models. Across all three rounds, participants identified the following three factors as the main contributors to their success (both in terms of direct giving to, and generating interest in, the project): family and friends (36%), personal networks (36%), and online networks (31%). These most frequently cited opinions are in synch with the results of the statistical analysis in that Facebook networks and sending out e-mails to social networks were among the most important drivers of a successful crowdfunding campaign.

**Table 11:** Factors mentioned by SciFund project creators that **helped** with project fundraising.

Factor	All rounds (n = 118)	Round 1 (n = 47)	Round 2 (n = 49)	Round 3 (n = 22)
Family and friends giving	43 (36%)	17 (36%)	18 (37%)	8 (36%)
Personal networks	43 (36%)	13 (28%)	23 (47%)	7 (32%)
Online networks	37 (31%)	20 (43%)	7 (14%)	10 (45%)
Effective video	13 (11%)	7 (15%)	4 (8%)	2 (9%)
Social relevance of project	8 (7%)	6 (13%)	2 (4%)	0
General SciFund publicity	5 (4%)	4 (9%)	0	1 (5%)
Small financial goal	4 (3%)	4 (9%)	0	0
National media	2 (2%)	2 (4%)	0	0
Tastemaker involvement	2 (2%)	2 (4%)	0	0
Local media	1 (1%)	1 (2%)	0	0
Luck	1 (1%)	1 (2%)	0	0
Merton effect	1 (1%)	0	1 (2%)	0
Rewards	1 (1%)	1 (2%)	0	0
Specific project goals	1 (1%)	1 (2%)	0	0

Respondents could mention multiple factors. N refers to number of completed surveys.

**Table 12:** Factors mentioned by SciFund project creators that **hurt** project fundraising.

Factor	All rounds (n = 118)	Round 1 (n = 47)	Round 2 (n = 49)	Round 3 (n = 22)
Blogging or social media (Facebook, Google+, Twitter) did not work for me	23 (19%)	10 (21%)	7 (14%)	6 (27%)
Did not promote enough	15 (13%)	8 (17%)	5 (10%)	2 (9%)
Had no online network or online media presence	14 (12%)	8 (17%)	4 (8%)	2 (9%)
Could not engage professional discipline or relevant organizations	10 (8%)	3 (6%)	3 (6%)	4 (18%)
Could not get press	10 (8%)	3 (6%)	6 (12%)	1 (5%)
Project focus or topic not good	8 (7%)	6 (13%)	2 (4%)	0
Tastemaker involvement not effective	7 (6%)	4 (9%)	3 (6%)	0
Friends and family would not donate	6 (5%)	4 (9%)	2 (4%)	0
Rewards were not a draw	5 (4%)	3 (6%)	1 (2%)	1 (5%)
Bad video or problems with video	4 (3%)	2 (4%)	1 (2%)	1 (5%)
Cold calls and reaching out to strangers not effective	4 (3%)	0	4 (8%)	0
Being faculty as opposed to student	1 (1%)	0	1 (2%)	0
Dollar goal too high	1 (1%)	1 (2%)	0	0
Male voice on video not effective	1 (1%)	1 (2%)	0	0
Timing of the campaign and national events	1 (1%)	0	0	1 (5%)

Respondents could mention multiple factors. N refers to number of completed surveys.

The other component of a successful campaign, according to the statistical analysis, is press contacts. However, this was not considered a key reason for success by the majority of participants. Less than 5% of the sample across the three rounds identified #SciFund publicity (4%), national media (2%), and local media (1%) as being important to their success.

Among the factors that did not work according to the participants, 19% of the sample thought that engaging their online networks (Facebook, Twitter, blogging, and Google) was unsuccessful. Related to this, 13% of the participants thought that they did not promote their project enough (to a variety of potential networks and press outlets). The third most cited factor considered to be unsuccessful was having a small or non-existent online network or social media presence. These impressions are in line with the statistical analysis in that the most frequent answers to this question were related to engaging social networks.

## Discussion

**Figure 8:**
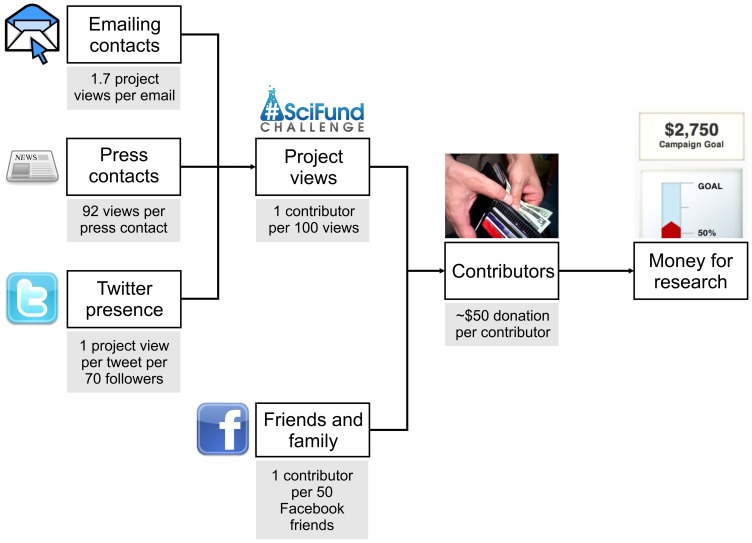
How online engagement leads to a crowdfunded research project.

Our analysis shows that engagement of broad audiences is the key to successful science crowdfunding. To engage, a scientist must first build an audience for their work, hopefully well before their crowdfunding campaign begins, such as through the Twitter and Facebook networks we quantified here. Once the crowdfunding begins, a scientist must then put effort into maintaining the connections between these networks and their science, such as through tweets or direct contact via email. Some activities, such as reaching out via the press, even accomplish the goals of both building a wider audience and connecting these audiences to a scientist's crowdfunding proposals all at the same time. Engagement via science communication then leads to research dollars by bringing people to view project pages. In turn, those views translate into contributions for new scientific work (Fig. 8; see Fig. S1 for a full path diagram with coefficients, and Figs. S2 and S3 for a similar visualization from round 1). In short, audience multiplied by outreach effort equals successful public engagement, and successful science crowdfunding.

### The Role of Audience

Our analyses show that the pathway to raising money via crowdfunding in science requires building a network of people interested in one's work and engaging that audience and additional members of the public interested in a specific project. This occurs largely before the crowdfunding campaign begins, and time invested in engagement yields a larger audience and proportionately greater funds raised. For example, our analyses suggested that Twitter and Facebook network size influences project success. While some of this did indeed come from family and friends, a scientist cannot grow these audiences. Rather, they must build other audiences, as reflected by contributions of Twitter, emails to supporting organizations, and direct contacts with the press. Additional forms of outreach to build one's scientific fan base not measured by our survey (e.g., involvement with museums, public lectures, TEDx talks, authoring popular science articles for a newspaper, media history, etc.) quite likely help in crowdfunding a project. These kinds of community engagement activities may facilitate access to local mailing lists as well as the likelihood of a press contact translating into an article. All of these forms of audience building demonstrate the importance of building and maintaining a consistent public presence for raising money through crowdfunding.

### Effort: You Are Not Shouting Into the Void

Having an audience alone is not enough to be successful. If a scientist launches a crowdfunding campaign, but doesn't tell anyone in their vast audience about it, that audience won't come. However, in the survey data, many scientists admitted to doubts that their efforts were successful. The quantitative data, in contrast, shows that while promotion of a crowdfunding project may at times feel like shouting into the void, the effort can and will lead to success. During a crowdfunding campaign, more effort – that is more tweets, more emails sent, more people in the press contacted - all led to higher funding. Crowdfunding takes effort. Informally, some successful participants reported spending ½–1 hour per day on outreach during their crowdfunding campaign period. Note that this is after the time-intensive process of producing crowdfunding materials, such as a short video, necessary to engage with a broad non-expert audience. These activities are different from the traditional grant-writing models that are comfortable for most scientists. Rather, these are the activities of a successful outreach program, but with the added benefit of research funding for the time invested.

### Differences between First and Subsequent Rounds

There were two main differences between our exploratory analysis of round one and the results of our confirmatory analysis in rounds two and three. First, blogging was not important in building an audience in rounds two and three. This may well reflect an artifact of participant self-selection. In round one, science crowdfunding was new, and many of our participants had a long history of engaging in online science outreach. Many were active bloggers with long-standing followings (authors' personal observations), sometimes built up over years (mean blog age = 28 months). In contrast, while many participants in later rounds had substantial Twitter audiences, they often did not have the long experience blogging (mean blog age = 14 months) despite having a relatively similar fraction of bloggers (51%, 35%, 50%, respectively).

The second difference between the rounds emerged due to differing methodology. Simply put, our Likert scale questions could not adequately capture effort in round 1. The shift to non-Likert questions regarding effort in rounds two and three allowed us to quantify a phenomenon we suspected was important given qualitative interviews, but had not been able to fully capture quantitatively.

### Moving Beyond the US$10K Barrier in Science Crowdfunding

Throughout #SciFund, we were commonly asked whether crowdfunding might someday serve as a replacement for traditional sources of funding. The amounts raised by the #SciFund projects were small compared to a typical National Science Foundation or National Institutes of Health grant. However, they are very much in line with initial crowdfunding efforts in many fields where crowdfunding is now a major source of revenue; a development period of a few years seems to be required for larger amounts to be raised via this method in any given field [44]. Indeed for #SciFund, there is evidence that the audience is growing. For example, the percentage of #SciFund projects meeting their goals increased each round (Figure S5), and after a recent fourth round (run on a different platform, Experiment.com, and hence not included here for analysis), scientists are now achieving a 62.5% success rate.

Furthermore, since the inception of #SciFund, several science crowdfunding projects have raised substantially more money than the most successful #SciFund projects. Two projects investigating the bacterial communities associated with humans each raised over US$300,000 [60], [61]. A project to launch a space telescope raised over US$1,000,000 [62]. The difference between these projects and #SciFund projects was rewards that directly involve citizens in the scientific process. Donors funding the two microbial projects at a certain minimum level had their very own bacterial communities analyzed by those projects. Funding the space telescope at high levels gave funders direct access to time on the telescope.

Examples of US$100K+ science crowdfunding efforts reinforce the basic lessons of our analyses. The scientists behind these high-earning crowdfunding campaigns also went to great lengths to promote their work. But more importantly, they went to extreme lengths to engage citizens in their scientific process. Audiences were captivated by taking part themselves in microbial and space research. They will likely be engaged with those scientific groups for years to come, potentially crowdfunding future projects.

### The Future of Crowdfunding for Science

Will crowdfunding replace traditional funding sources? No. At the bare minimum, science crowdfunding provides a tangible financial reward for outreach, enabling access to untapped pools of research funds while removing the “waste-of-time” stigma of outreach [47]. Moreover, it opens up a new pool of funds for pilot or high-risk projects, allowing a scientist to later leverage their engaged audience alongside preliminary data for larger pools of funds. However, for projects that engage heavily with the public (i.e., provide opportunities for citizen science) or emerge from labs who are deeply engaged with the community around them, crowdfunding may provide a truly alternative funding mechanism for many kinds of research projects.

A common concern is that crowdfunding will only be viable for projects with lowest common denominator public appeal, such as projects with charismatic large animals (“panda bear science”), a human health aspect, or some other element that has populist appeal, regardless of the scientific importance of the project. Many successful #SciFund Challenge projects were on topics that are not normally considered popular with the public, however (e.g., statistics, little known invertebrates, etc.). This is not to say that all projects will have equal appeal, but that persistent engagement can build an audience for many kinds of projects. The key to creating an engaging proposal is communicating why the project sparks your passion, and why should it matter to your audience.

### Making Crowdfunding Part of a Research Group and University's Funding Portfolio

Our work suggests a clear path forward for individual researchers who wish to fund a portion of their research group's work via crowdfunding. We suggest that researchers should begin by cultivating an audience for their work over time. This can be through a variety of avenues: become active in local public science efforts, foster connections with relevant non-governmental organizations with their own audiences, launch a public science blog (potentially with collaborators), build a Twitter following, and search out as many ways to easily communicate your science to as broad an audience as possible. The skills for running a campaign are identical to those needed to build an audience in the first place. A scientist who has built an audience will therefore have an easier experience running their campaign. When it comes time to crowdfund a project, these are the sources that can be tapped for research funding; this “fan base” will already be invested and engaged in your work. More importantly, once you have crowdfunded your work, maintain the connections with your funders. Keep them apprised of progress. Keep them involved with the process and results of your science. This constant contact has two benefits: first, it should enable more successful repeat crowdfunding, and potentially higher levels of future funding. Second, and more importantly, it will yield direct social benefits by connecting progressively more people to science.

In these times of stagnant traditional science funding, every piece of external funding helps labs and universities move forward. Ultimately, if universities want to take advantage of crowdfunding dollars, academic culture must embrace science engagement, in contrast to the current climate of devaluing outreach in university hiring and promotion policies [25], [27], [28], [29], [30], [31], [32], [38], [39], [40], [41], [42], [43], [47], [63]. To be competitive in the new and dynamic crowdfunding environment, universities must find ways to develop and enrich policies and practices that foster active outreach and engagement by their faculty.

#SciFund illustrates that fostering a strong connection between science and society within the culture of academia can benefit both universities and scientists financially. But the benefits of creating an academic climate that encourages science outreach are greater than a new source of research funding. Outreach and engagement create public science literacy [64], new arenas of public support for science, and new connections between scientists and the world that they are trying to understand.

## Supporting Information

Figure S1 The pathway of interactions leading to money raised for projects in round two and three. Diagram shows the relationships between different variables in our analyses. Only those relationships that explained significant amounts of variation are included (LR ÷^2^ test p≤0.05). Coefficients represent linear relationships and are in the units of variables described. Sample size varies between each analysis represented in the diagram below due to differences in respondent behaviour and the exclusion or inclusion of outlier data.
(PDF)

Figure S2 How online engagement leads to a crowdfunded research project based on results from round 1.
(PDF)

Figure S3 The pathway of interactions leading to money raised for projects. Diagram shows the relationships between different variables in our analyses. Only those relationships that explained significant amounts of variation are included (LR χ^2^ test p≤0.05). Coefficients represent linear relationships and are in the units of variables described with one exception. The relationship between Facebook “Likes” and post-goal page views is exponential, and is shown as such. Sample size varies between each analysis represented in the diagram below due to differences in respondent behavior and the exclusion or inclusion of outlier data.
(PDF)

Figure S4 Component-residual plots showing the relationship between pre-goal page views, press contacts, number of people emailed, and effort times engagement on Twitter in rounds two and three. Tweet reach = number of Twitter followers × number of tweets. Press2 = number of people contacted in the press. Email = number of people contacted via email.
(PDF)

Figure S5 Percent of projects hitting 100% of their funding goal over the first four rounds of the #SciFund Challenge.
(PDF)

Table S1 Snapshot of money raised by projects on Cancer Research UK. Table shows money raised by the 43 projects that were live on Cancer Research UK on May 9, 2012 (data collected on this date from Cancer Research UK website: http://myprojects.cancerresearchuk.org/projects).
(DOCX)

Table S2 Survey given to round one #SciFund participants. Numerous questions that required a response on a Likert scale (e.g., questions 47–55, 63–71) were changed to require specific numerical responses for the round two and three survey instrument. In addition, the round one dates associated with questions 72–74 were changed to the appropriate round two and three dates for their respective surveys.
(DOCX)
