# Supplementary material for: To Crowdfund Research, Scientists Must Build an Audience for Their Work
Source: PLoS One. 2014 Dec 10;9(12):e110329. doi: 10.1371/journal.pone.0110329 (PMC4262210; doi:10.1371/journal.pone.0110329)

Figure S1: **The pathway of interactions leading to money raised for projects in round two and three.** Diagram shows the relationships between different variables in our analyses. Only those relationships that explained significant amounts of variation are included (LR χ^2 test $p \leq 0.05$). Coefficients represent linear relationships and are in the units of variables described. Sample size varies between each analysis represented in the diagram below due to differences in respondent behavior and the exclusion or inclusion of outlier data.

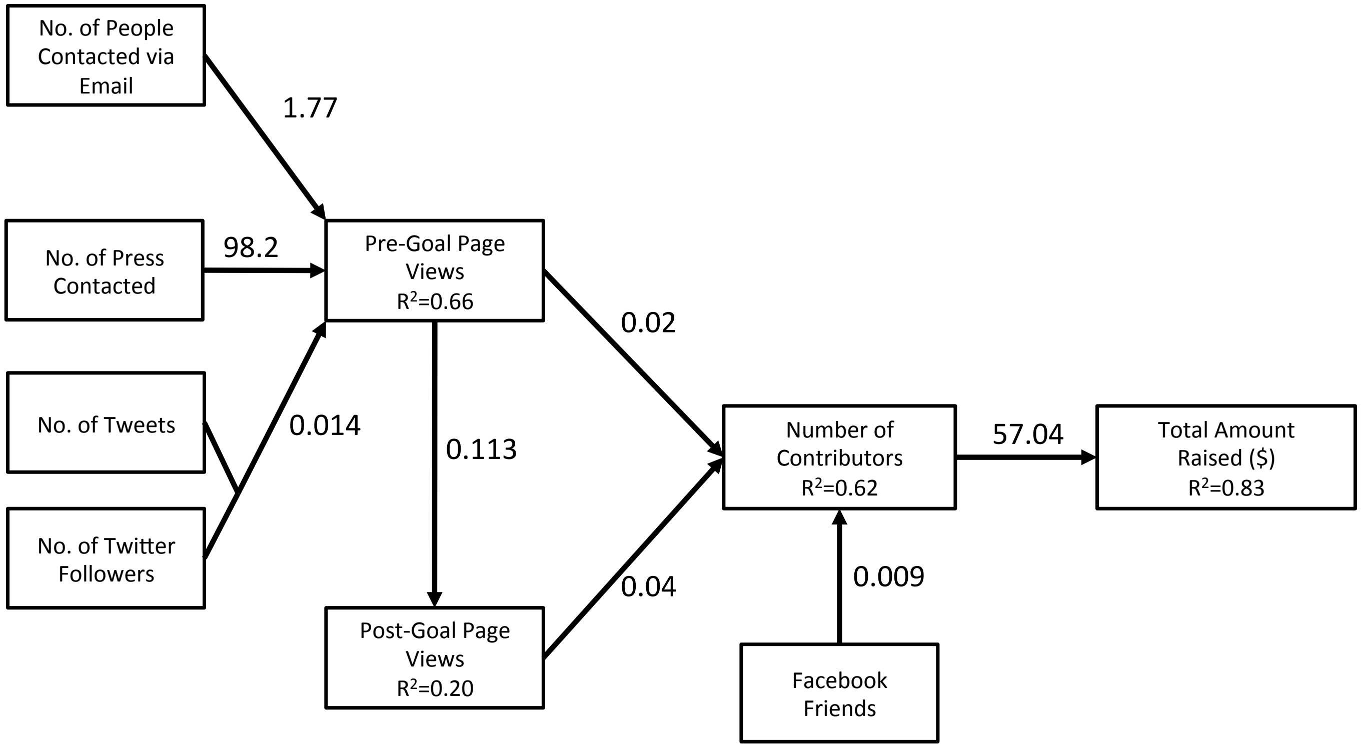

Supplement: Figure S1 — The pathway of interactions leading to money raised for projects in round two and three. Diagram shows the relationships between different variables in our analyses. Only those relationships that explained significant amounts of variation are included (LR ÷2 test p≤0.05). Coefficients represent linear relationships and are in the units of variables described. Sample size varies between each analysis represented in the diagram below due to differences in respondent behaviour and the exclusion or inclusion of outlier data. (PDF) [file pone.0110329.s001.pdf]
