# Supplementary material for: To Crowdfund Research, Scientists Must Build an Audience for Their Work
Source: PLoS One. 2014 Dec 10;9(12):e110329. doi: 10.1371/journal.pone.0110329 (PMC4262210; doi:10.1371/journal.pone.0110329)

Figure S2: **Our view of how online engagement leads to a crowdfunded research project based on results from round 1.**

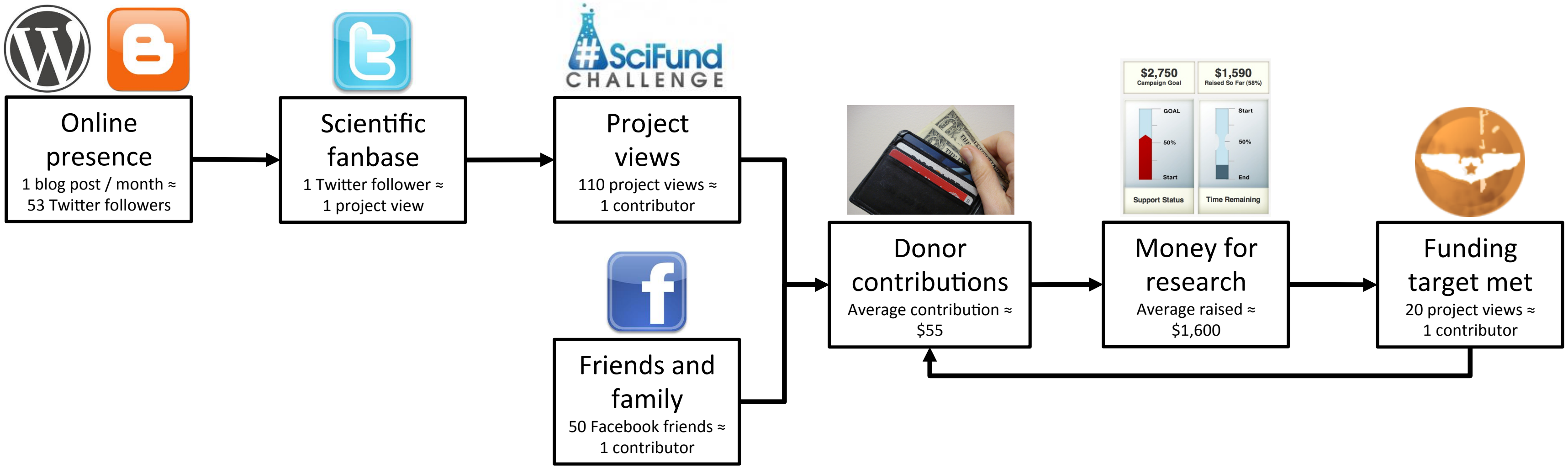

Supplement: Figure S2 — How online engagement leads to a crowdfunded research project based on results from round 1. (PDF) [file pone.0110329.s002.pdf]
