# Supplementary material for: To Crowdfund Research, Scientists Must Build an Audience for Their Work
Source: PLoS One. 2014 Dec 10;9(12):e110329. doi: 10.1371/journal.pone.0110329 (PMC4262210; doi:10.1371/journal.pone.0110329)

Figure S4: **Component-residual plots showing the relationship between pre-goal page views, press contacts, number of people emailed, and effort * engagement on Twitter in rounds two and three.** Tweet Reach = number of Twitter followers * number of tweets. Press2 = number of people contacted in the press. email = number of people contacted via email.

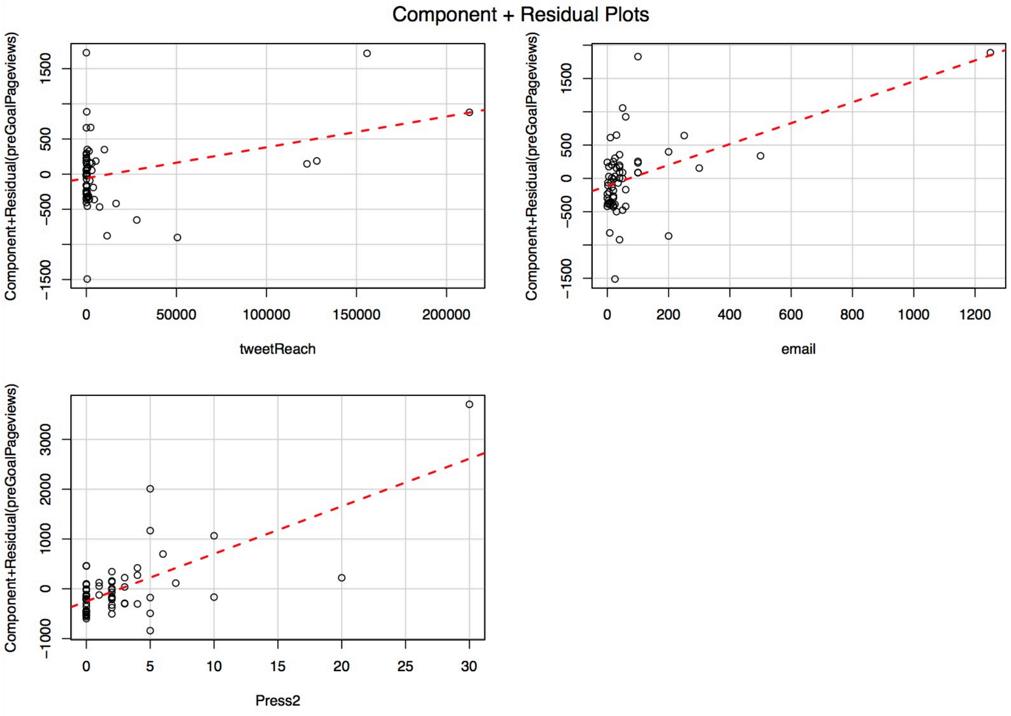

Supplement: Figure S4 — Component-residual plots showing the relationship between pre-goal page views, press contacts, number of people emailed, and effort times engagement on Twitter in rounds two and three. Tweet reach = number of Twitter followers × number of tweets. Press2 = number of people contacted in the press. Email = number of people contacted via email. (PDF) [file pone.0110329.s004.pdf]
