# Supplementary material for: To Crowdfund Research, Scientists Must Build an Audience for Their Work
Source: PLoS One. 2014 Dec 10;9(12):e110329. doi: 10.1371/journal.pone.0110329 (PMC4262210; doi:10.1371/journal.pone.0110329)
Supplement: Figure S5 — Percent of projects hitting 100% of their funding goal over the first four rounds of the #SciFund Challenge. (PDF) [file pone.0110329.s005.pdf]

Figure S5: **Changes in the percentage of projects hitting 100% of their funding goal over the four rounds of the #SciFund Challenge run to date.**

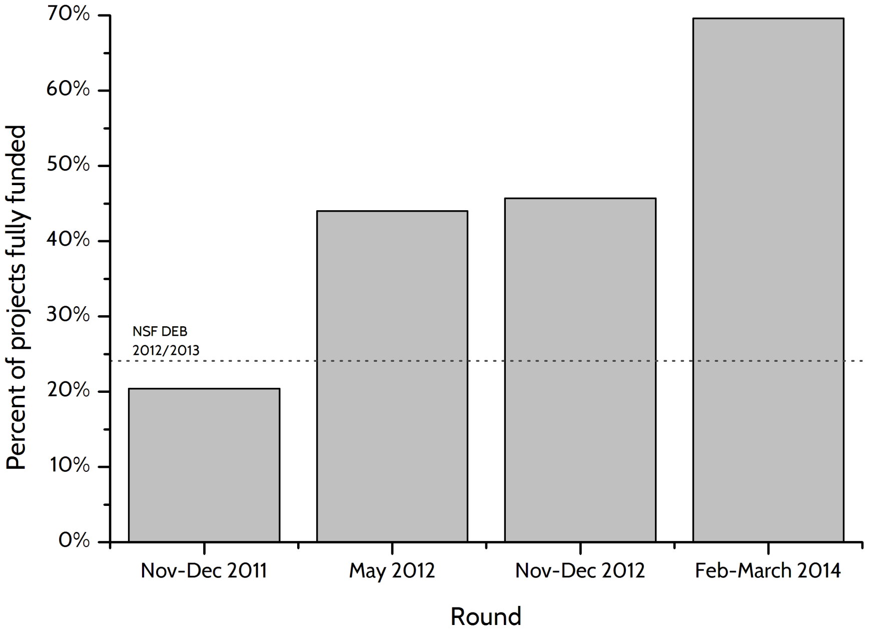

NSF DEB full proposal success rate from
https://nsfdeb.wordpress.com/2013/12/30/deb-numbers-fy2013-wrap-up/
