# Supplementary material for: To Crowdfund Research, Scientists Must Build an Audience for Their Work
Source: PLoS One. 2014 Dec 10;9(12):e110329. doi: 10.1371/journal.pone.0110329 (PMC4262210; doi:10.1371/journal.pone.0110329)
Supplement: Table S1 — Snapshot of money raised by projects on Cancer Research UK. Table shows money raised by the 43 projects that were live on Cancer Research UK on May 9, 2012 (data collected on this date from Cancer Research UK website: http://myprojects.cancerresearchuk.org/projects). (DOCX) [file pone.0110329.s006.docx]

|  |  |  |  |  |
| --- | --- | --- | --- | --- |

**Table S1. Snapshot of money raised by projects on Cancer Research UK.** Table shows money raised by the 43 projects that were live on Cancer Research UK on May 9, 2012 (data collected on this date from Cancer Research UK website:<http://myprojects.cancerresearchuk.org/projects>).

| **Funds raised (£)** | **Financial goal (£)** |
| --- | --- |
| 172,462 | 180,000 |
| 104,707 | 200,000 |
| 104,605 | 104,000 |
| 84,021 | 109,000 |
| 77,286 | 110,000 |
| 71,505 | 150,000 |
| 66,879 | 71,000 |
| 66,015 | 100,000 |
| 59,180 | 180,000 |
| 56,098 | 220,000 |
| 54,859 | 100,000 |
| 51,000 | 51,000 |
| 49,075 | 63,000 |
| 47,921 | 105,000 |
| 39,590 | 135,000 |
| 39,081 | 50,000 |
| 37,092 | 60,000 |
| 36,486 | 78,000 |
| 33,905 | 180,000 |
| 26,113 | 237,000 |
| 25,450 | 50,000 |
| 21,164 | 100,000 |
| 20,174 | 20,000 |
| 20,000 | 20,000 |
| 16,576 | 52,500 |
| 15,833 | 68,000 |
| 15,699 | 20,000 |
| 15,000 | 15,000 |
| 12,361 | 70,000 |
| 11,417 | 70,000 |
| 10,599 | 200,000 |
| 8,156 | 35,000 |
| 8,082 | 340,000 |
| 7,709 | 15,000 |
| 6,980 | 30,000 |
| 5,391 | 40,000 |
| 4,975 | 100,000 |
| 1,345 | 25,000 |
| 175 | 60,000 |
| 125 | 20,000 |
| 0 | 20,000 |
| 0 | 20,000 |
| 0 | 120,000 |
