# Supplementary material for: To Crowdfund Research, Scientists Must Build an Audience for Their Work
Source: PLoS One. 2014 Dec 10;9(12):e110329. doi: 10.1371/journal.pone.0110329 (PMC4262210; doi:10.1371/journal.pone.0110329)
Supplement: Table S2 — Survey given to round one #SciFund participants. Numerous questions that required a response on a Likert scale (e.g., questions 47–55, 63–71) were changed to require specific numerical responses for the round two and three survey instrument. In addition, the round one dates associated with questions 72–74 were changed to the appropriate round two and three dates for their respective surveys. (DOCX) [file pone.0110329.s007.docx]

|  |  |  |  |  |
| --- | --- | --- | --- | --- |

**Table S1. Snapshot of money raised by projects on Cancer Research UK.** Table shows money raised by the 43 projects that were live on Cancer Research UK on May 9, 2012 (data collected on this date from Cancer Research UK website:<http://myprojects.cancerresearchuk.org/projects>).

| **Funds raised (£)** | **Financial goal (£)** |
| --- | --- |
| 172,462 | 180,000 |
| 104,707 | 200,000 |
| 104,605 | 104,000 |
| 84,021 | 109,000 |
| 77,286 | 110,000 |
| 71,505 | 150,000 |
| 66,879 | 71,000 |
| 66,015 | 100,000 |
| 59,180 | 180,000 |
| 56,098 | 220,000 |
| 54,859 | 100,000 |
| 51,000 | 51,000 |
| 49,075 | 63,000 |
| 47,921 | 105,000 |
| 39,590 | 135,000 |
| 39,081 | 50,000 |
| 37,092 | 60,000 |
| 36,486 | 78,000 |
| 33,905 | 180,000 |
| 26,113 | 237,000 |
| 25,450 | 50,000 |
| 21,164 | 100,000 |
| 20,174 | 20,000 |
| 20,000 | 20,000 |
| 16,576 | 52,500 |
| 15,833 | 68,000 |
| 15,699 | 20,000 |
| 15,000 | 15,000 |
| 12,361 | 70,000 |
| 11,417 | 70,000 |
| 10,599 | 200,000 |
| 8,156 | 35,000 |
| 8,082 | 340,000 |
| 7,709 | 15,000 |
| 6,980 | 30,000 |
| 5,391 | 40,000 |
| 4,975 | 100,000 |
| 1,345 | 25,000 |
| 175 | 60,000 |
| 125 | 20,000 |
| 0 | 20,000 |
| 0 | 20,000 |
| 0 | 120,000 |

**Table S2**. **Survey given to round one #SciFund participants.** Numerous questions that required a response on a Likert scale (e.g., questions 47-55, 63-71) were changed to require specific numerical responses for the round two and three survey instrument. In addition, the round one dates associated with questions 72-74 were changed to the appropriate round two and three dates for their respective surveys.

| 1. Name |
| --- |
| 2. Age |
| 3. Gender |
| 4. #SciFund Challenge Project Name |
| 5. Ethnicity |
| 6. Current position |
| 7. Your institution |
| 8. What is the discipline of your highest degree? |
| 9. What is your highest degree you have completed? |
| 10. How many hours did you spend on your last grant proposal? |
| 11. Over the past five years, what approximate percentage of your grant proposals have been funded? |
| 12. Do you use Facebook? |
| 13. If you use Facebook, how many Facebook friends do you have now? |
| 14. Do you use Twitter? |
| 15. If you use Twitter, how many Twitter followers do you have now? |
| 16. If you use Twitter, for how many months have you been using it? |
| 17. Do you have a science-oriented blog? |
| 18. If you have a science-oriented blog, how many times a month on average do you post to it? |
| 19. If you have a science-oriented blog, for how many months has it existed? |
| 20. In the past twelve months, how many times have you: [Written a science-oriented article for a general interest news platform (print or online)] |
| 21. In the past twelve months, how many times have you: [Given a public talk about science intended for an audience of non-scientists] |
| 22. In the past twelve months, how many times have you: [Been interviewed by a journalist for a science story] |
| 23. If you are engaged in other types of social media, please describe. |
| 24. Did you have any experience with video editing prior to participating in the #SciFund Challenge? |
| 25. If you do have video experience, how many videos have you worked on (no matter how short)? |
| 26. How many hours did you spend: [Writing project text] |
| 27. How many hours did you spend: [Working on your video] |
| 28. How many hours did you spend: [Preparing images] |
| 29. How many hours did you spend: [Preparing and sending out project rewards] |
| 30. How many hours did you spend: [Making comments to other #SciFund Challenge projects on the wiki] |
| 40. To what degree did you change your #SciFund Challenge project in response to feedback on the wiki or RocketHub? [Text] |
| 41. To what degree did you change your #SciFund Challenge project in response to feedback on the wiki or RocketHub? [Images] |
| 42. To what degree did you change your #SciFund Challenge project in response to feedback on the wiki or RocketHub? [Video] |
| 43. To what degree did you change your #SciFund Challenge project in response to feedback on the wiki or RocketHub? [Rewards] |
| 44. Did you shorten your video? |
| 45. How many times did you change your video after posting it? |
| 46. How many hours did you spend promoting your project? |
| 47. What methods did you use to spread the word about your project (1 - not at all, 5 - a great deal)? [E-mail] |
| 48. What methods did you use to spread the word about your project (1 - not at all, 5 - a great deal)? [Facebook] |
| 49. What methods did you use to spread the word about your project (1 - not at all, 5 - a great deal)? [Twitter] |
| 50. What methods did you use to spread the word about your project (1 - not at all, 5 - a great deal)? [Google +] |
| 51. What methods did you use to spread the word about your project (1 - not at all, 5 - a great deal)? [Blogging on your own blog] |
| 52. What methods did you use to spread the word about your project (1 - not at all, 5 - a great deal)? [Guest blogging on others' blogs] |
| 53. What methods did you use to spread the word about your project (1 - not at all, 5 - a great deal)? [One-on-one conversations] |
| 54. What methods did you use to spread the word about your project (1 - not at all, 5 - a great deal)? [Public talks/appearances] |
| 55. What methods did you use to spread the word about your project (1 - not at all, 5 - a great deal)? [Communicating with journalists/bloggers] |
| 56. If you used other methods to spread the word about your project, please describe. |
| 57. Did you spread the word about your project via (1 - not at all, 5 - a great deal) [Personal connections] |
| 58. Did you spread the word about your project via (1 - not at all, 5 - a great deal) [Professional connections] |
| 59. What do you feel worked to get your project funded? |
| 60. What do you feel did not work to get your project funded? |
| 61. Do you feel that your #SciFund Challenge campaign was a success? Why or why not? |
| 62. How many hours did you spend promoting OTHER #SciFund projects? |
| 63. What methods did you use to spread the word about OTHER #SciFund projects (1 - not at all, 5 - a great deal)? [E-mail] |
| 64. What methods did you use to spread the word about OTHER #SciFund projects (1 - not at all, 5 - a great deal)? [Facebook] |
| 65. What methods did you use to spread the word about OTHER #SciFund projects (1 - not at all, 5 - a great deal)? [Twitter] |
| 66. What methods did you use to spread the word about OTHER #SciFund projects (1 - not at all, 5 - a great deal)? [Google +] |
| 67. What methods did you use to spread the word about OTHER #SciFund projects (1 - not at all, 5 - a great deal)? [Blogging on your own blog] |
| 68. What methods did you use to spread the word about OTHER #SciFund projects (1 - not at all, 5 - a great deal)? [Guest blogging on others' blogs] |
| 69. What methods did you use to spread the word about OTHER #SciFund projects (1 - not at all, 5 - a great deal)? [One-on-one conversations] |
| 70. What methods did you use to spread the word about OTHER #SciFund projects (1 - not at all, 5 - a great deal)? [Public talks/appearances] |
| 71. What methods did you use to spread the word about OTHER #SciFund projects (1 - not at all, 5 - a great deal)? [Communicating with journalists/bloggers] |
| 72. How much effort did you make to spread the word about your project during: [November 1-15] |
| 73. How much effort did you make to spread the word about your project during: [November 16-30] |
| 74. How much effort did you make to spread the word about your project during: [December 1-15] |
| 75. If you stopped promoting your project before the end of the #SciFund campaign period, please describe why. |
| 76. What percentage of the contributors to your project do you personally know? |
| 77. How many press or new media figures did you notify about your project? |
| 78. How many live events (not via computer) did you use to promote your project? |
| 79. If you hosted live events in support of your project, what was the average attendance at these events? |
| 80. Do you feel that the #SciFund Challenge as a whole was a success? Why or why not? |
